# Clinical significance of B7-H3 expression in circulating CD4^+^CD25^high^ T cells, CD14^+^ monocytes, and plasma for the progression of HIV infection

**DOI:** 10.1186/s12879-023-08411-9

**Published:** 2023-07-10

**Authors:** Jun-Chi Xu, Hui Chen, Ping Xu, Xin-Ran You, Geng-chao Zhu, Fei Gao

**Affiliations:** 1 The Affiliated Suzhou Hospital of Nanjing Medical University, 26 Daoqian Road, Suzhou, Jiangsu P. R. China; 2 Suzhou Municipal Hospital, 26 Daoqian Road, Suzhou, Jiangsu P. R. China; 3 The Fifth People’s Hospital of Suzhou, China. 10, Guangqian Road, Suzhou, Jiangsu 215000 P. R. China

**Keywords:** B7-H3, HIV infection, CD4^+^CD25^high^ T cells, Monocytes, Progression

## Abstract

**Background:**

B7-H3 is an important immune checkpoint molecule that plays a negative role in immune regulation. This study was aimed to explore B7-H3 expression in HIV-infected patients and its clinical significance.

**Methods:**

To explore the expression and clinical significance of B7-H3 in HIV-infected patients, we investigated the B7-H3 expression pattern and the correlation of B7-H3 expression with clinical parameters of HIV-infected patients with different levels of CD4^+^ T cells. To assess the role of B7-H3 in regulating the function of T cells in HIV infection, we performed a proliferation assay and T cell function test in vitro.

**Results:**

B7-H3 expression in HIV-infected patients was significantly higher than that in healthy controls. mB7-H3 expression on CD4^+^CD25^high^ T cells and CD14^+^ monocytes increased with disease progression. mB7-H3 expression on CD4^+^CD25^high^ T cells and monocytes was negatively correlated with lymphocyte count, CD4^+^T cell count, and positively correlated with HIV viral load in HIV-infected patients. when the number of CD4^+^ T cells in HIV-infected patients was ≥ 200/µL, sB7-H3 and mB7-H3 expression levels on CD4^+^CD25^high^ T cells and monocytes were negatively correlated with lymphocyte count, CD4^+^T cell count. sB7-H3 and mB7-H3 expression on monocytes were positively correlated with HIV viral load. B7-H3 inhibited the proliferation of lymphocytes and the secretion of IFN-γ in vitro, especially the ability of CD8^+^ T cells to secrete IFN-γ.

**Conclusions:**

B7-H3 played an important negative regulatory role in anti-HIV infection immunity. It could be used as a potential biomarker for the progression of HIV infection and a novel target for the treatment of HIV infection.

**Supplementary Information:**

The online version contains supplementary material available at 10.1186/s12879-023-08411-9.

## Background

Acquired immunodeficiency syndrome (AIDS) caused by human immunodeficiency virus (HIV) infection has become one of the most devastating infectious diseases in human history [1]. According to WHO statistics, 36.9 million persons live with HIV/AIDS, and 940 000 persons died of HIV-related illnesses worldwide in 2017. Antiviral therapy is currently the main method of controlling HIV infection. Although retroviral therapy can reduce the replication rate of HIV, it is not possible to completely eradicate the virus or restore the function of virus-specific cluster of differentiation (CD) 4^+^T cells and CD8^+^T cells. Finding an effective detection marker and therapeutic target for HIV infection is of great value.

HIV mainly invades the human immune system, including CD4^+^T lymphocytes, monocytes, macrophages, and dendritic cells. The main clinical manifestation is that the number of CD4^+^ T lymphocytes is continuously decreasing, which ultimately leads to defects in human cellular immune function, causing various opportunistic infections and tumors [2, 3]. HIV antigen-specific CD4^+^T lymphocytes and antigen-specific cytotoxic T lymphocytes play an important role in anti-HIV responses. Rebuild the immune function of HIV-infected patients is essential for successful anti-viral therapy.

Different stages of HIV infection are often accompanied by excessive activation and exhaustion of the immune system. The costimulatory signaling pathway is critical for the activation of T cells. A treatment strategy involving the regulation of the costimulatory molecular signaling pathway provides new methods for improving the immune function of HIV-infected patients. The B7:CD28 superfamily is a group of costimulatory molecules involved in the regulation of T cell activation, tolerance and autoimmunity. B7 molecules include programmed cell death 1 ligand 1 (PD-L1) and B7 homolog 3 (B7-H3), and CD28 receptors include programmed cell death 1 (PD-1). Studies have shown that PD-1 and PD-L1 are related to immune function defects in HIV infection [4, 5]. HIV infection causes virus-specific T cells, especially CD8^+^ T cells, to upregulate the expression of PD-1, resulting in decreased proliferation, cytokine release and ability to kill cells, which leads to a “functional exhaustion” state [6, 7]. Blocking the PD-1/PD-L1 pathway can increase the secretion of cytokines such as interferon-γ (IFN-γ), tumor necrosis factor-α (TNF-α) and interleukin (IL)-2, and restore the cytotoxic function of CD8^+^ T cells [8]. Blocking the PD-1/PD-L1 pathway can decrease the HIV viral load in the HIV-infected mouse model, suggesting that blocking the PD-1/PD-L1 pathway may be a potential treatment for HIV-infected patients [9]. Blocking the PD-1/PD-L1 pathway enhances CD4^+^ T cell and CD8^+^ T cell functions during Simian immunodeficiency virus infection [10].

B7-H3 is also an important costimulatory ligand that plays a negative role in immune regulation. Analysis of B7-H3 expression on antigen presenting cells and immunoregulatory cells is essential for exploring the role of B7-H3 in disease. B7-H3 is available in both soluble (sB7-H3) and membrane-bound (mB7-H3) forms. Our previous study found that B7-H3 was increased in patients with Hepatitis B Virus (HBV) infection and might contribute to the progression of HBV infection by triggering inhibitory signals in effector T cells [11, 12]. B7-H3 is also highly expressed in breast cancer, ovarian cancer, non-small cell lung cancer, prostate cancer and other tumors [13–16]. In pancreatic ductal adenocarcinoma, CAR-T targeted therapy with B7-H3 as a target can significantly inhibit tumor growth [17]. These studies indicated the important role of B7-H3 in anti-infection immunity and antitumor immunity. Research has shown that HIV-infected lung cancer patients have higher B7-H3 tumor expression compared to HIV-uninfected controls [18]. However, there is no research report on the expression and role of B7-H3 in immune cells of HIV-infected patients. CD4^+^CD25^high^ T cells and monocytes are immunoregulatory cells which play key roles in HIV and HBV infection [11, 19, 20]. in this study, we aimed to explore sB7-H3 and B7-H3 expression on CD4^+^CD25^high^ T cells and monocytes in HIV-infected patients and its clinical significance.

## Methods

### Clinical samples

Blood samples from HC (n = 20) and patients with HIV infection (n = 42) were collected. Healthy donors were identified to be without HIV-1, HBV, HCV, and HDV infection or other known diseases. All patients were HIV positive, in line with the HIV/AIDS national diagnostic criteria of People’s Republic of China. Patients with autoimmune diseases, other immunodeficiencies, tuberculosis, hepatitis and other infectious diseases, and malignant tumors were excluded. To analyse the role of B7-H3 in the different course of HIV infection, we divided HIV-infected patients into 2 groups according to CD4^+^ T cell count. The 42 patients were divided into CD4^+^ T cells ≥ 200/µL group (HIV- infected patients with high CD4^+^ T cells) and CD4^+^ T cells < 200/µL group (HIV- infected patients with low CD4^+^ T cells). Our study was approved by the Ethics Committee of The Fifth People’s Hospital of Suzhou. Informed consent was obtained from all patients involved in this study.

### Enzyme-linked immunosorbent assay

Peripheral blood samples were collected with plasma preparation tubes. The tubes were then centrifuged at 3,000 rpm for 15 min, and the serum was collected. The levels of sB7-H3 and IFN-γ in the serum were analyzed with enzyme-linked immunosorbent assay (ELISA) kits (BlueGene, Co., Ltd., Shanghai, China) according to the manufacturer’s instructions.

### Flow cytometry analysis

To determine mB7-H3 expression in the peripheral blood, anti-CD3-APC, anti-CD4-PerCP, anti-CD25-FITC, anti-CD14-FITC, and anti-IFN-γ-PE were purchased from BD Pharmingen (San Jose, CA); B7-H3-PE was purchased from BioLegend (San Diego, CA). For the detection of membrane surface molecules, 50 µL of fresh whole blood from the healthy donors or patients was incubated with antibodies (10 µL) for 15 min, lysed with red blood cell lysis buffer, then incubated with phosphate-buffered saline, and eventually detected by BD FACS Aria with BD FACS Diva software support (San Jose, CA). For the detection of IFN-γ, PBMCs of HIV-infected patients were stimulated with PMA (10 ng/ml) and ionomycin (1 µg/ml) for 4 h and incubated for the last 3 h with brefeldin A (10 µg/ml). The cells were transferred to a U-bottom plate, stained with surface marker antibodies in HBSS containing 1% FCS, fixed with 2% formaldehyde and permeabilized with 0.5% saponin. The cells were stained with anti- IFN-γ, washed with phosphate-buffered saline, fixed and eventually detected with a BD FACS Aria with BD FACS Diva (San Jose, CA, USA) software support. Data were analyzed by FlowJo (San Carlos, CA).

### Laboratory examination

Forty-two HIV-infected patients were enrolled and venous blood was collected during hospitalization. BD FACS (San Jose, CA) was used to detect the Frequency of T cell subsets. Lymphocyte count was collected from routine blood test data (XN1000, SYSMEX, Japan). HIV-RNA was quantified with the COBAS AmpliPrep/COBAS TaqMan HIV Test Kit (Roche, Germany).

### Cell proliferation assay

Peripheral blood mononuclear cells (PBMCs) from HIV-infected patients were seeded into 96-well plates with anti-CD3 antibody (100 ng/mL) (eBioscience, USA) + the recombinant human IgG1 Fc protein (20 ng/mL), and anti-CD3 antibody (100 ng/mL) + recombinant human B7-H3 Fc chimera protein (20 ng/mL) (R&D Systems, USA), then cultured at 37 °C, and 5% CO2. Cell proliferation ability was examined after 24, 48, and 72 h by counting the cells.

### Statistical analysis

Statistical analysis was performed using SPSS 22.0 software (SPSS Inc., Chicago, IL). Nonparametric tests (Kruskal-Wallis-test), Spearman correlation test and repeated measures analysis of variance were applied to compare statistical evaluations among groups and for correlation analysis. A two-tailed p < 0.05 was considered statistically significant.

## Results

### Investigating sB7-H3 expression and mB7-H3 expression on CD4^+^CD25^high^ T cells and CD14^+^ monocytes in HIV-infected patients

**Fig. 1:**
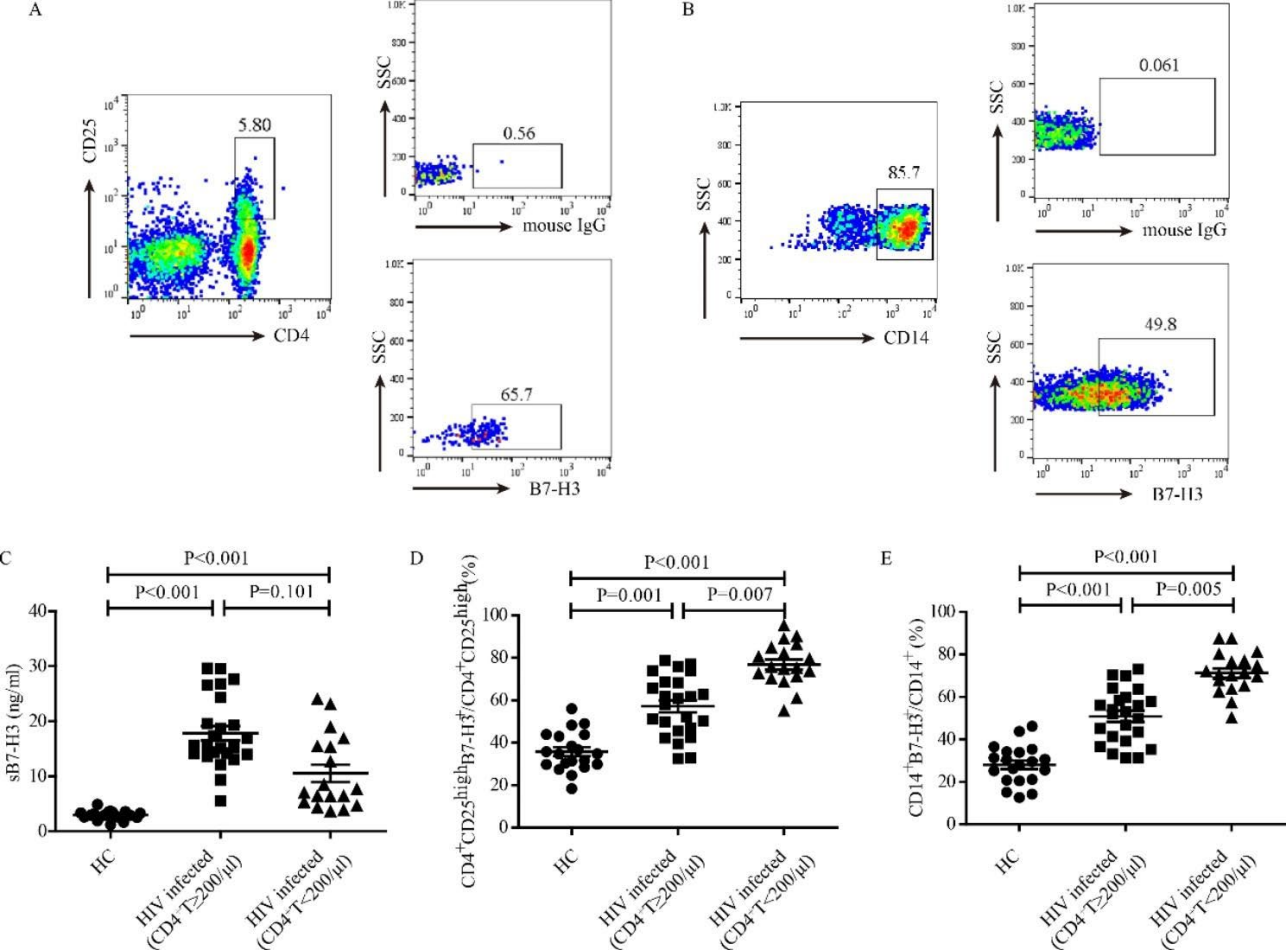
The expression profiles of B7-H3 in HIV-infected patients. (**A**, **B**) The gating strategies and representative results of B7-H3 expression on circulating CD4^+^CD25^high^ T cells and CD14^+^ monocytes. (**C**-**E**) Expression pattern of B7-H3 in peripheral blood, circulating CD4^+^CD25^high^ T cells, and circulating CD14^+^ monocytes

This study detected the B7-H3 expression pattern in HIV-infected patients with different levels of CD4^+^ T cells. Gating strategies for the CD4^+^CD25^high^B7-H3^+^ and CD14^+^B7-H3^+^ populations are shown in Fig. 1A and B. sB7-H3 expression in the plasma of HIV-infected patients with low and high CD4^+^ T cells increased profoundly compared with that of healthy control donors (HC) (both P < 0.001) (Fig. 1C). mB7-H3 expression on CD4^+^CD25^high^ T cells of HIV-infected patients with low and high CD4^+^ T cells significantly increased compared with that of HC (P = 0.001, and P < 0.001, respectively). The percentage of CD4^+^CD25^high^B7-H3^+^T cells in HIV-infected patients with low CD4^+^ T cells was higher than that in HIV-infected patients with high CD4^+^T cells (P = 0.007) (Fig. 1D). There was a significant increase in the frequency of circulating CD14^+^B7-H3^+^ monocytes in HIV-infected patients with low and high CD4^+^ T cells compared with the HC group (both P < 0.001). The percentage of CD14^+^B7-H3^+^ monocytes in HIV-infected patients with low CD4^+^ T cells was also higher than that in HIV-infected patients with high CD4^+^ T cells (P = 0.005) (Fig. 1E). This study also analyzed B7-H3 expression of the bulk CD4^+^ population during the progression of HIV infection. It showed that B7-H3 expression on CD4^+^T cells increased with disease progression (sFig 1).

### Analyzing the correlation between sB7-H3 and clinical parameters of HIV- infected patients

**Fig. 2:**
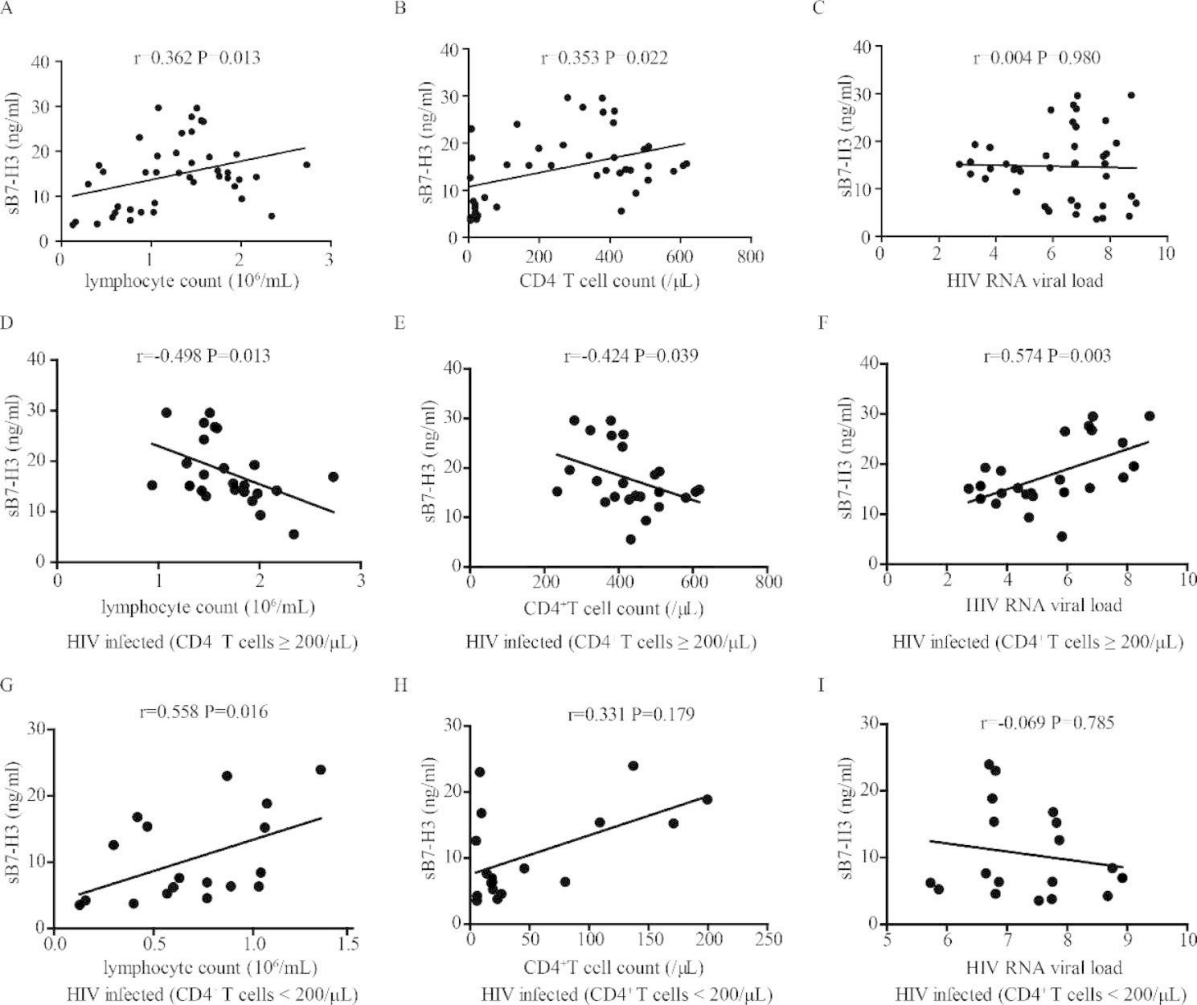
The correlation between sB7-H3 expression and clinical parameters of HIV-infected patients. (**A**-**C**) The correlation between sB7-H3 expression and lymphocyte count, CD4^+^ T cell count, and HIV viral load in HIV-infected patients. (**D**-**F**) The correlation between sB7-H3 expression and lymphocyte count, CD4^+^ T cell count, and HIV viral load in HIV-infected patients with a high frequency of CD4^+^ T cells. (**G**-**I**) The correlation between sB7-H3 expression and lymphocyte count, CD4^+^ T cell count, and HIV viral load in HIV-infected patients with a low frequency of CD4^+^ T cells

sB7-H3 was positively correlated with lymphocyte count (r = 0.362, P = 0.013) and CD4+ T cell count (r = 0.353, P = 0.022) in HIV-infected patients (Fig. 2A, B). In HIV-infected patients with high CD4^+^ T cells, sB7-H3 was negatively correlated with lymphocyte count (r=-0.496, P = 0.013) and CD4^+^T cells (r=-0.424, P = 0.039) and positively correlated with HIV viral load (r = 0.574, P = 0.003) (Fig. 2D-F). In HIV-infected patients with low CD4^+^ T cells, sB7-H3 was positively correlated with lymphocyte count (r = 0.558, P = 0.016), but had no correlation with the CD4^+^T cell count (r = 0.331, P = 0.179) or HIV viral load (r=-0.069, P = 0.785) (Fig. 2G-I).

### The correlation between mB7-H3 expression on CD4+CD25high T cells and clinical parameters of HIV- infected patients

**Fig. 3:**
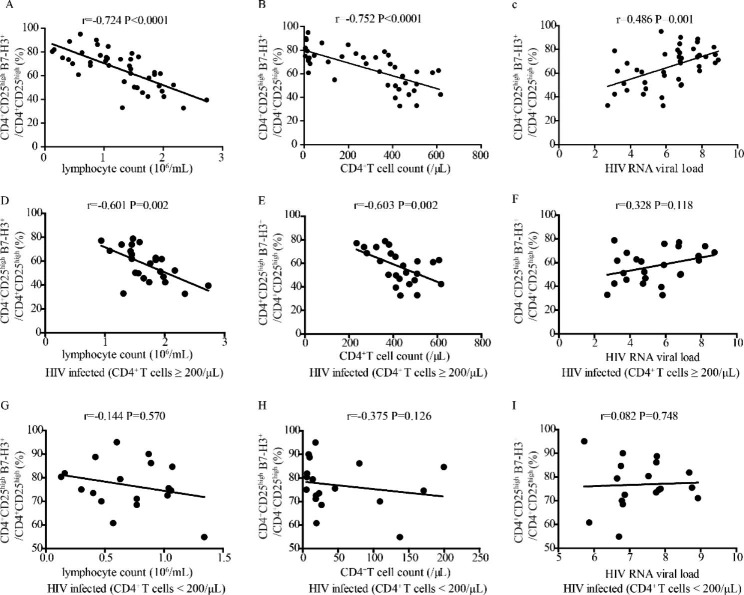
The correlation between mB7-H3 expression on CD4^+^CD25^high^ T cells and clinical parameters of HIV-infected patients. (**A**-**C**) The correlation between mB7-H3 expression on CD4^+^CD25^high^ T cells and lymphocyte count, CD4^+^T cell count, and HIV viral load in HIV-infected patients. (**D**-**F**) The correlation between mB7-H3 expression on CD4^+^CD25^high^ T cells and lymphocyte count, CD4^+^T cell count, and HIV viral load in HIV-infected patients with a high frequency of CD4^+^T cells. (**G**-**I**) The correlation between mB7-H3 expression on CD4^+^CD25^high^ T cells and lymphocyte count, CD4^+^T cell count, and HIV viral load in HIV-infected patients with a low frequency of CD4^+^T cells

mB7-H3 expression on CD4^+^CD25^high^ T cells was negatively correlated with lymphocyte count (r=-0.724, P < 0.0001), CD4^+^T cell count (r=-0.752, P < 0.0001), and positively correlated with HIV viral load (r = 0.486, P = 0.001) in HIV-infected patients (Fig. 3A-C).

mB7-H3 expression on CD4^+^CD25^high^ T cells of HIV-infected patients with high CD4^+^ T cells was negatively correlated with Lymphocyte count (r=-0.601, P = 0.002) and CD4^+^T cell count (r=-0.603, P = 0.002) but had no correlation with HIV viral load (r = 0.328, P = 0.118) (Fig. 3D-F). There was no correlation between mB7-H3 expression on CD4^+^CD25^high^ T cells and the clinical parameters of HIV-infected patients with low CD4^+^ T cells (Fig. 3G-I).

### The correlation between mB7-H3 expression on monocytes and clinical parameters of HIV- infected patients

**Fig. 4:**
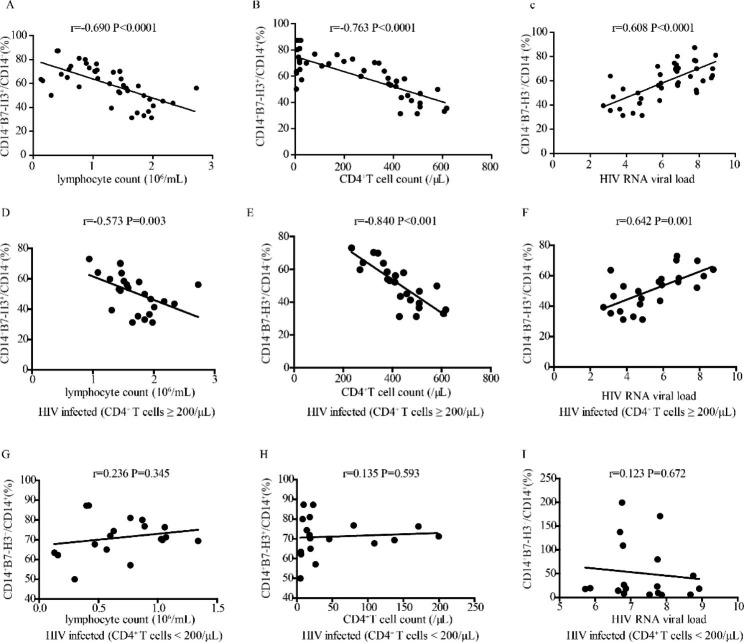
The correlation between mB7-H3 expression on CD14^+^ monocytes and clinical parameters of HIV-infected patients. (**A**-**C**) The correlation between mB7-H3 expression on monocytes and lymphocyte count, CD4^+^T cell count, and HIV viral load in HIV-infected patients. (**D**-**F**) The correlation between mB7-H3 expression on CD14^+^ monocytes and lymphocyte count, CD4^+^T cell count, and HIV viral load in HIV-infected patients with a high frequency of CD4^+^ T cells. (**G**-**I**) The correlation between mB7-H3 expression on CD14^+^ monocytes and lymphocyte count, CD4^+^T cell count, and HIV viral load in HIV-infected patients with a low frequency of CD4^+^ T cells

mB7-H3 expression on CD14^+^ monocytes was negatively correlated with lymphocyte count (r=-0.690, P < 0.0001), CD4^+^ T cell count (r=-0.763, P < 0.0001), and positively correlated with HIV viral load (r = 0.608, P < 0.0001) in HIV-infected patients (Fig. 4A-C).

The frequency of CD14^+^B7-H3^+^monocytes in HIV-infected patients with high CD4^+^ T cells was negatively correlated with lymphocyte count (r=-0.573, P = 0.003) and CD4^+^ T cell count (r=-0.840, P < 0.001) and positively correlated with HIV viral load (r = 0.642, P = 0.001) (Fig. 4D-F). There was no correlation between mB7-H3 expression on CD14^+^ monocytes and the clinical parameters of HIV-infected patients with low CD4^+^ T cells (Fig. 4G-I).

### B7-H3 inhibited T cell function in HIV patients

**Fig. 5:**
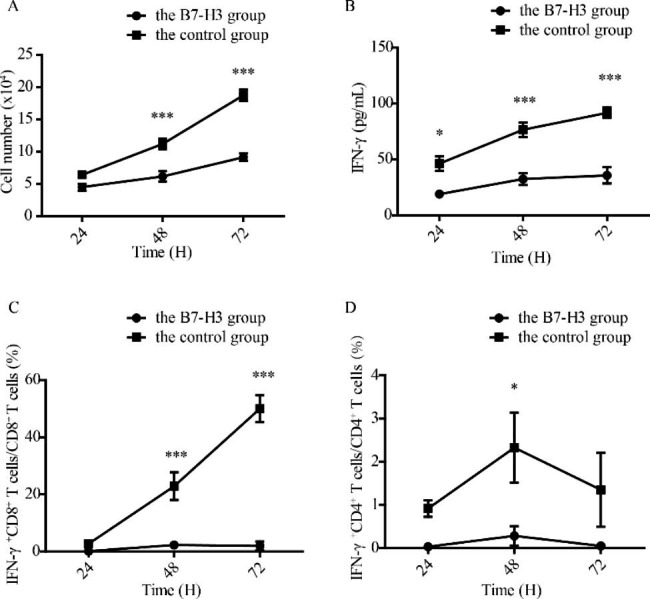
The role of B7H3 in regulating the function of T cells in HIV infection. (**A**) B7-H3 inhibited the proliferation of T cells. (**B**) B7-H3 inhibited the secretion of IFN-γ. (**C**) B7-H3 inhibited the secretion of IFN-γ in CD8^+^ T cells. (**D**) B7-H3 inhibited the secretion of IFN-γ in CD4^+^ T cells. * P < 0.05, ** P < 0.01, ***P < 0.001

To assess the role of B7-H3 in regulating the function of T cells in HIV infection, a proliferation assay and an IFN-γ secretion assay were performed. Compared with the control group, the B7-H3 group had a lower rate of proliferation at 48 hand 72 h (P > 0.05, P < 0.001, and P < 0.001, respectively) (Fig. 5A). There was a significant increase in the secretion of IFN-γ in the B7-H3 group compared with the control group at 24 h, 48 h, and 72 h (P < 0.05, P < 0.001, and P < 0.001, respectively) (Fig. 5B). To investigate the effects of B7-H3 on the function of CD4^+^ T cells and CD8^+^ T cells, we examined the ability of CD4^+^ T cells and CD8^+^ T cells to secrete IFN-γ after the addition of B7-H3. The results showed that B7-H3 inhibited the secretion of IFN-γ from CD4^+^ T cells and CD8^+^ T cells (Fig. 5C and D).

## Discussion

T lymphocytes play a crucial role in controlling HIV infection and viral replication. They can directly kill HIV-infected cells through cytotoxicity and inhibit HIV replication in infected cells by secreting cytokines [21]. However, there are many dysfunctions in T cells in vivo after HIV infection. Among them, T cell exhaustion mediated by negative costimulatory molecules is one of the factors that affects the body’s immune level and antiviral ability. Studies have shown that PD-1/PD-L1and T cell immunoglobulin and mucin-domain containing-3 expression are elevated in HIV-infected patients, leading to defects in T cell function [22, 23]. B7-H3 is an important inhibitory costimulatory molecule that plays an essential role in infection immunity and antitumor immunity. However, the expression pattern and the clinical significance of BT-H3 in HIV-infected patients are still unknown.

To clarify the clinical significance of B7-H3 over the course of HIV infection, we detected the expression pattern of B7-H3 and its correlation with patient clinical parameters at different stages of HIV progression. The results showed that the B7-H3 expression in HIV-infected patients was significantly higher than that in healthy controls. mB7-H3 expression on CD4^+^CD25^high^ T cells and CD14^+^ monocytes increased with disease progression (Fig. 1C-E). We analyzed the association of B7-H3 expression with lymphocyte count, CD4^+^T cell count, and HIV viral load to explore the relationship between B7-H3 expression and the immune system and disease progression in HIV-infected patients. The results showed that mB7-H3 expression on CD4^+^CD25^high^ T cells (Fig. 3A-C) and monocytes (Fig. 4A-C) was negatively correlated with Lymphocyte count, CD4^+^T cell count, and positively correlated with HIV viral load in HIV-infected patients. The positive correlation between B7-H3 expression and HIV viral load indicated that upregulation of B7-H3 is associated with HIV infection. To analyse the role of B7-H3 in the different course of HIV infection, we divided HIV infected patients into 2 groups according to CD4^+^ T cell count. The same correlations between mB7H3 expression and clinical parameters in HIV-infected patients were maintained in HIV-infected patients with CD4^+^ T cells ≥ 200/µL (Figs. 3D-F and 4D-F ). But in HIV-infected patients with CD4^+^ T cells < 200/µL, the above correlations disappeared (Figs. 3G-I and 4G-I). It may due to the complete collapse of the patient’s immune function in the advanced stage. Based on these results, mB7-H3 expression on CD4^+^CD25^high^ T cells and monocytes could be a potential biomarker for the progression of HIV.

sB7-H3 concentration was negatively correlated with lymphocyte count, CD4^+^T cell count, and positively correlated with HIV viral load in HIV-infected patients with CD4^+^T cells ≥ 200/µL (Fig. 2D-F). These correlations were not maintained in HIV-infected patients overall (Fig. 2A-C) and HIV-infected patients with CD4^+^ T cells < 200/µL (Fig. 2G-I). The immune system collapsed in the advanced stage of the disease. The excessive apoptosis of immune cells influenced the correlation analysis of sB7-H3 concentration and above pathological parameters. These suggested sB7-H3 concentration could be a potential biomarker for the progression of HIV in the early HIV infection period.

To further explore the role of B7-H3 in regulating the function of T cells in HIV infection, we observed the effect of B7-H3 on lymphocyte proliferation and IFN-γ secretion ability. B7-H3 inhibited lymphocyte proliferation (Fig. 5A) and the secretion of IFN-γ, especially the ability of CD8^+^ T cells to secrete IFN-γ (Fig. 5B-D). The combination of co-stimulatory molecules of T cell and ligands on antigen presenting cells is essential for T cell activation [24]. The above results suggested B7-H3 could bind to the receptor on HIV-specific CD4^+^T cells and CD8^+^T cells, and suppress the immune function of effector cells. CD8^+^ T cells, which play an important role in antiviral responses, are the most important immune cells in HIV viral clearance. Inhibition of B7-H3 on lymphocyte proliferation and IFN-γ secretion by CD8^+^ T may be one of the reasons that the immune function of HIV-infected patients is difficult to rebuild and the virus cannot be effectively eliminated. In combination with the correlation between B7-H3 expression and the disease progresses, lymphocyte count, CD4^+^T cells count and HIV viral load, we could find that B7-H3 could be used as a potential biomarker for the progression of HIV infection and related to immune function defects in HIV infection.

This study has its limitations. On the one hand, further studies among the population with larger sample size are necessary to evaluate the possibility of B7-H3 to be the biomarker for the progression of HIV infection and the B7-H3 expression between infected and non-infected cells. On the other hand, this study initially analyzed the effect of B7-H3 on T cells in HIV infection by in vitro experiments; however, there is still no further verification in animal models to explore the therapeutic effect of blocking the B7-H3 signaling pathway on HIV infection and to further study the role and mechanism of B7-H3 in HIV infection.

## Conclusion

In summary, B7-H3 was highly expressed in HIV-infected patients and increased with disease progression. Its expression was related to lymphocyte count, CD4^+^T cell count and HIV virus load. B7-H3 inhibited the proliferation of lymphocytes and the secretion of IFN-γ in vitro. The negative signaling pathway mediated by B7-H3 may be one of the reasons that the immune function of HIV-infected patients is difficult to rebuild and the virus cannot be effectively eliminated. B7-H3 is expected to become a potential biomarker for the progression of HIV infection and a novel target for the treatment of HIV infection.

## Electronic supplementary material

Below is the link to the electronic supplementary material.

Supplementary Material 1

## Data Availability

The datasets generated and analysed during the current study are available in the figshare repository, [https://figshare.com/articles/figure/Raw_data_of_patients_with_HIV_infection_xlsx/19687842].

## Declarations

**Ethics approval and consent to participate**.

This study was approved by the Ethics Committee of The Fifth People’s Hospital of Suzhou. Informed consent was obtained from all patients/ their parents/ legal guardians which were involved in this study. All procedures performed in studies involving human participants were in accordance with the ethical standards of the institutional and national research committee and with the Declaration of Helsinki.

**Consent for publication**.

## Abbreviations

AIDS: Acquired immunodeficiency syndrome
HIV: human immunodeficiency virus
CD: cluster of differentiation
PD-L1: programmed cell death 1 ligand 1
PD-1: programmed cell death 1
B7-H3: B7 homolog 3
sB7-H3: soluble B7-H3
mB7-H3: membrane-bound B7-H3
IFN-γ: interferon-γ
TNF-α: tumor necrosis factor-α
IL: interleukin
HC: healthy control
HBV: Hepatitis B Virus
