# Supplementary figures and images for: Clinical significance of B7-H3 expression in circulating CD4+CD25high T cells, CD14+ monocytes, and plasma for the progression of HIV infection
Source: BMC Infect Dis. 2023 Jul 10;23:462. doi: 10.1186/s12879-023-08411-9 (PMC10334634; doi:10.1186/s12879-023-08411-9)

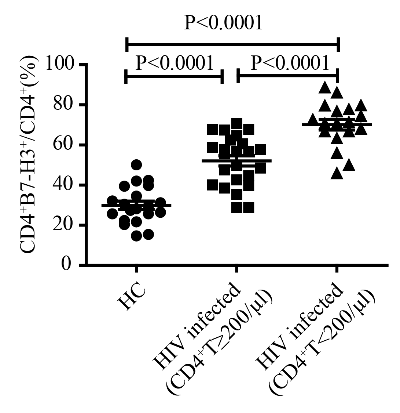


sFIG. 1. B7-H3 expression on CD4+T cells increased with disease progression

Supplement: Supplementary file 1 — Supplementary Material 1 [file 12879_2023_8411_MOESM1_ESM.docx]
